# Molecular characterization of carbapenem-resistant and carbapenem-sensitive *Acinetobacter baumannii* isolates from an intensive care unit in Ningbo, China

**DOI:** 10.3389/fmicb.2025.1646319

**Published:** 2025-09-03

**Authors:** Xinwei Chen, Yanye Tu, Feng Wang, Hong Li, Yujie Zhao, Min Jiang, Hui Gao, Wei Zhang

**Affiliations:** Department of Clinical Laboratory, The Affiliated Li Huili Hospital of Ningbo University, Ningbo, Zhejiang, China

**Keywords:** carbapenem-resistant *Acinetobacter baumannii*, intensive care unit, efflux pump inhibitor, virulence genes, resistance genes

## Abstract

**Objective:**

This study aims to examine the variations in resistance genes, virulence genes, and drug susceptibility between carbapenem-resistant and carbapenem-susceptible *Acinetobacter baumannii* (CR-AB and CS-AB). Additionally, it seeks to assess the influence of efflux pump inhibitors on drug susceptibility, in order to provide new antimicrobial treatment strategies for CR-AB infections in the intensive care unit (ICU).

**Methods:**

A retrospective study was undertaken involving 39 *A. baumannii* (*A. baumannii*) strains isolated from the ICU of Li Huili Hospital, affiliated with Ningbo University, during the period from January to December 2023. Of these strains, 18 were classified as CR-AB and 21 as CS-AB. The minimum inhibitory concentrations (MICs) of commonly employed clinical antibiotics, polymyxin B, tigecycline, and ceftazidime/avibactam, were assessed using the microdilution method. The alterations in MICs of ceftazidime/avibactam for CR-AB isolates were evaluated before and after the incorporation of the efflux pump inhibitor phenylalanine-arginine-β-naphthylamine (PAβN). Whole genome sequencing (WGS) was conducted to elucidate the differences in resistance and virulence genes, and phenotypic validation of these virulence gene differences was performed utilizing the *Galleria mellonella* larvae model.

**Results:**

The CR-AB isolates demonstrated substantial resistance to ceftazidime, cefepime, ceftriaxone, ampicillin/sulbactam, tobramycin, gentamicin, and levofloxacin, while exhibiting moderate resistance to trimethoprim-sulfamethoxazole and amikacin. Conversely, the CS-AB isolates remained susceptible to all the aforementioned commonly utilized clinical antibiotics. Antimicrobial susceptibility testing indicated that 2.56% of the 39 *A. baumannii* strains displayed resistance to polymyxin B, with no resistance detected against tigecycline. The minimum inhibitory concentration (MIC) ranges for polymyxin B, tigecycline, and ceftazidime/avibactam were 0.125 μg/mL to 4 μg/mL, 0.25 μg/mL to 1 μg/mL, and 2/4 μg/mL to 256/4 μg/mL, respectively. PAβN was observed to reduce the MIC values of ceftazidime/avibactam against CR-AB in a concentration-dependent manner. Relative to the CS-AB isolates, the CR-AB isolates not only exhibited a more complex resistance gene profile but also showed greater diversity and abundance in their virulence gene profiles. The survival rate of CR-AB isolates was significantly lower in the *G. mellonella* larvae model, indicating that CR-AB strains from the ICU in Ningbo have evolved toward increased virulence and resistance.

**Conclusion:**

The CR-AB isolates from the ICU in Ningbo demonstrate heightened resistance and virulence traits in comparison to the CS-AB isolates. The application of the efflux pump inhibitor PAβN markedly increases the susceptibility of CR-AB to ceftazidime/avibactam.

## Introduction

*Acinetobacter baumannii* (*A. baumannii*), a significant opportunistic pathogen associated with nosocomial infections, has demonstrated a rising prevalence on a global scale in recent years. A longitudinal study conducted in Saudi Arabia indicated that the clinical isolation rate of *A. baumannii* increased from 7% in 2018 to 13% in 2022 (Alharbi et al., 2025). Owing to its multidrug resistance, robust environmental adaptability, high transmission potential, significant virulence, and elevated mortality rate, alongside the limitations of existing preventive and control measures, this pathogen has been designated by the World Health Organization (WHO) as a priority resistant pathogen. It represents a significant challenge to global public health that necessitates immediate resolution (Helen et al., 2008; Ming-Feng and Chung-Yu, 2014; Morris et al., 2019; da Fonseca et al., 2021; Aswin et al., 2024). Molecular epidemiological research has demonstrated that *A. baumannii* has evolved into clonal strains characterized by multidrug resistance and increased virulence. This evolution is attributed to the acquisition of carbapenemase-encoding genes, such as *bla*_OXA-23_, which exhibits a detection rate of up to 78.5% based on surveillance data from Portugal. Additionally, the acquisition of virulence factors, including the capsular type KL120, has been observed (Domingues et al., 2024). Genomic analyses conducted in Eastern China and South Korea have verified that isolates of carbapenem-resistant *Acinetobacter baumannii* (CR-AB) frequently harbor a diverse array of resistance and virulence genes (Zhu et al., 2022; Park et al., 2023). Furthermore, the molecular epidemiological analysis employing core genome multilocus sequence typing (cgMLST) conducted by Hungarian researchers has markedly enhanced the precision in identifying associations between antibiotic resistance genes and virulence genes (Hummel et al., 2024). Over the past decade, *A. baumannii* has risen as an important pathogen in patients who are vulnerable and critically ill. The predominant clinical manifestations of *A. baumannii* infections encompass bloodstream infections, pneumonia, urinary tract infections, and skin and soft tissue infections. The mortality rate associated with ventilator-associated pneumonia (VAP) due to *A. baumannii* ranges from 40 to 70% (Garnacho-Montero et al., 2005), whereas the mortality rate for bloodstream infections lies between 28 and 43% (Chopra et al., 2013). The total attributable mortality rate approaches 35% (Cavallo et al., 2023).

In clinical practice, the management of *A. baumannii* infections frequently poses substantial challenges, especially with the emergence of CR-AB, which has considerably restricted the range of available antimicrobial therapies (Choi and Kim, 2024). Recent epidemiological surveillance data indicate a sustained rise in the prevalence of CR-AB isolates in clinical specimens (Rhythm and Dinesh, 2025), with this trend being especially pronounced among patients in the Intensive Care Unit (ICU) (Schechner et al., 2024; Tian et al., 2024). It is important to highlight that these prevalent CR-AB strains not only demonstrate extensive resistance profiles but also exhibit an increasing trend in virulence (Chukamnerd et al., 2022), thereby significantly heightening the risk of nosocomial infections and complicating clinical management in ICU patients.

This study focuses on the scarcity of molecular epidemiological data regarding CR-AB in Ningbo by analyzing 39 *A. baumannii* strains from ICU clinical samples collected at our hospital between January and December 2023. By combining whole genome sequencing (WGS), resistance profiling, and analysis of pathogenic phenotypes, this research provides the inaugural comprehensive description of the molecular epidemiological characteristics of ICU-derived CR-AB and carbapenem-susceptible *A. baumannii* (CS-AB) in this area. Notably, from the perspective of efflux pump inhibition, this study contributes novel scientific evidence pertinent to the antimicrobial treatment of ICU-associated CR-AB infections.

## Materials and methods

### Bacterial strain collection and selection

A retrospective selection of 39 *A. baumannii* strains, excluding duplicate isolates, was conducted using specimens collected from the ICU of Li Huili Hospital, affiliated with Ningbo University, between January and December 2023. In accordance with the Clinical and Laboratory Standards Institute (CLSI) guidelines (CLSI, 2023), Carbapenem resistance in *A. baumannii* is defined by an MIC of imipenem or meropenem that is ≥4 μg/mL. We identified 18 CR-AB strains and 21 CS-AB strains in this study, which received approval from the Medical Ethics Committee of Li Huili Hospital, Ningbo Medical Center (Approval No.: Li Huili Hospital Ethics Review 2022 Research No. 282).

### Bacterial identification and drug sensitivity testing

We identified *A. baumannii* using the VITEK-2 Compact system (bioMérieux, France) and confirmed it with the EXS3600 automated microbiological mass spectrometry system (Zybio, China). The microdilution broth method was used to test the susceptibility of various commonly used clinical antibiotics, including ceftazidime, cefepime, ceftriaxone, imipenem, ampicillin/sulbactam, amikacin, gentamicin, tobramycin, trimethoprim-sulfamethoxazole, levofloxacin, polymyxin B, tigecycline, and ceftazidime/avibactam (Lot DZ1548; Wenzhou Kangtai Technology Co., Ltd.). The protocol for antimicrobial susceptibility testing of frequently used antibiotics, via the microdilution broth method, is as follows: A bacterial suspension with turbidity equivalent to the 0.5 McFarland standard was prepared using fresh colonies from overnight cultures. A 10 μL portion of this bacterial suspension was moved to 2 mL of Mueller-Hinton broth, mixed well, and then 100 μL of the diluted suspension was added to the appropriate antimicrobial susceptibility plates. Incubation of the plates occurred at 37°C for 16 to 18 h. The MIC was assessed by evaluating bacterial growth or inhibition on susceptibility plates. According to the CLSI guidelines, the MIC breakpoints for typical antibiotics and polymyxin B were assessed (CLSI, 2023), with a polymyxin B MIC of ≥ 4 μg/mL indicating resistance. Although there are no established MIC breakpoints for *A. baumannii* for tigecycline and ceftazidime/avibactam, a tigecycline MIC of ≥ 8 μg/mL was considered resistant based on laboratory experience and published studies (Liu et al., 2022). For testing antimicrobial susceptibility, quality control strains such as *Escherichia coli* ATCC 25922 and *Pseudomonas aeruginosa* ATCC 27853 were sourced from the National Clinical Testing Center under the Ministry of Health.

### Efflux pump inhibition assay

We selected fresh colonies derived from overnight cultures that exhibited a ceftazidime/avibactam MIC of 64/4 μg/mL and prepared them into a bacterial suspension with a turbidity equivalent to 0.5 McFarland standard. This suspension was subsequently diluted 50-fold using autoclaved nutrient broth. The autoclaved nutrient broth served as the solvent for ceftazidime, avibactam, and the efflux pump inhibitor phenylalanine-arginine-β-naphthylamine (PAβN) (Shanghai Yuan Ye Biotechnology Co., Ltd.). The ceftazidime was prepared in a gradient of concentrations: 1024, 512, 256, 128, 64, 32, 16, 8, 4, 2, 1, and 0.5 μg/mL, while avibactam was maintained at a constant concentration of 16 μg/mL, and PAβN was tested at concentrations of 800, 400, 200, and 100 μg/mL. The experimental setup was organized into groups based on varying PAβN concentrations. For each group, 50 μL of each of the four PAβN concentrations and 50 μL of each of the 12 ceftazidime concentrations were combined in pairs and dispensed into a 96-well plate, with each combination replicated three times. In each well, 50 μL of avibactam at a concentration of 16 μg/mL and 50 μL of autoclaved nutrient broth were introduced. The resulting final concentrations in each well were as follows: PAβN at 200, 100, 50, and 25 μg/mL, and ceftazidime at 256, 128, 64, 32, 16, 8, 4, 2, 1, 0.5, 0.25, and 0.125 μg/mL. The concentration of avibactam was maintained at 4 μg/mL across all wells. The experimental setup also included a positive control group and a group containing only PAβN. Using a SpectraMax Plus 384 microplate reader (Molecular Devices, United States), we monitored bacterial growth by measuring OD600 every 2 h during 37°C incubation.

### Whole genome sequencing and analysis

The WGS of 39 *A. baumannii* strains was conducted using the Illumina NovaSeq platform (Illumina, Inc., United States), with raw reads being quality-filtered through fastp v0.20.1 (Chen et al., 2018). SOAP denovo, SPAdes, and Abyss software were used to assemble the sequences, and CISA software was employed to integrate the assembly results from these tools. The initial assembly was further gap-closed using gapclose software to obtain the final assembly. The study employed stringent BLASTP alignments (*E*-value cutoff ≤1 × 10^−5^) for sequence homology searches, with unified annotation thresholds set at ≥40% sequence identity and ≥40% coverage. For each query gene, only the top-scoring alignment result was retained for functional annotation. Genome sequence annotation was performed using Prokka 1.11. The core-genome content of the strain collection was determined using Roary v3.12.0, and the estimates of recombination within clades identified in the phylogeny were conducted with Gubbins v2.3.1 using default settings. The maximum likelihood phylogenetic tree was constructed by IQ-TREE v2.2.2 with 1,000 bootstrap replicates. The tree file was visualized using iTOL v5. To visualize the clonal relationships and potential temporal dynamics among *A. baumannii* isolates, we constructed Minimum Spanning Trees (MSTs) based on core genome SNP data. Initially, core genome SNP information for all isolates was extracted from previously obtained whole-genome sequencing data. Subsequently, these SNP data were processed using PhyloViz software v2.0 to generate the MST networks. As a set of modular tools, the MOB-suite was used to reconstruct and type plasmids. Distribution of mobilizable plasmid clusters analyzed by the MOB-suite pipeline.

### Preparation of the *Galleria mellonella* larva infection model

For the experiment, we employed the *Galleria mellonella* larva infection model as described by previous studies (Ménard et al., 2021). We selected healthy *G. mellonella* larvae (sourced from Huiyude, Tianjin) with a weight range of 250–350 mg, characterized by a white body surface, absence of black spots, and robust mobility. We performed preliminary experiments to determine the optimal bacterial concentration for larval infection. Using three bacterial suspensions (10^7^, 10^6^, and 10^5^ CFU/mL), we systematically assessed larval mortality rates. We defined mortality by two criteria: (1) visible black discoloration and (2) absence of response to mechanical stimulation. Our results showed that 10^5^ CFU/mL produced the most consistent infection outcomes. Therefore, we selected this concentration for all subsequent experimental procedures. In the formal experimental procedure, each isolate was used to inoculate a cohort of 10 larvae. A micropipette was used to precisely inject 10 μL of bacterial suspension into the side base of the second-to-last abdominal segment of each larva, with precautions to avoid leakage after injection. After inoculation, the larvae were transferred to sterile Petri dishes and incubated at 37°C in a light-free setting. Observations were systematically conducted at 6-h intervals during the initial 24-h period, followed by 12-h intervals from 24 to 72 h, and 24-h intervals from 72 to 144 h. A survival curve was subsequently generated. The experimental design incorporated a negative control group, which received a saline solution, and a positive control group, which was inoculated with *A. baumannii* ATCC-AB5075, obtained from the National Clinical Medical Testing Center.

### Statistical analysis

Statistical analyses were conducted utilizing SPSS software version 22.0. Categorical data comparisons were executed employing the *χ*^2^ test or Fisher’s exact test. Differences in the prevalence of virulence gene carriers and the MIC values of ceftazidime/avibactam for *A. baumannii* were assessed using the Mann–Whitney *U* test. Survival analysis was undertaken using the Log-rank (Mantel–Cox) test. A *p*-value < 0.05 was deemed to indicate statistical significance.

## Results

### Basic clinical data of isolates

From January to December 2023, the ICU at the Affiliated Li Huili Hospital of Ningbo University gathered 39 *A. baumannii* isolates. Among these, 18 isolates were identified as CR-AB, and 21 as CS-AB, resulting in a CR-AB detection rate of 46.15%. Gender, age, and specimen type showed no statistically significant differences between the CR-AB and CS-AB isolates (Table 1). Analysis of the specimen sources revealed that sputum samples constituted the largest proportion for both CR-AB and CS-AB isolates (CR-AB: 66.67%, 12/18; CS-AB: 66.67%, 14/21). Other specimen types included blood, pleural (peritoneal) fluid, bronchoalveolar lavage fluid, cerebrospinal fluid, urine, and secretions.

**Table 1:** Constitution in gender, age group, and specimen sources of CR-AB and CS-AB.

Item		Number [percentage (%)]		*χ* ^2^	*p-*value
		CR-AB	CS-AB		
Gender	Male	14(77.78%)	14(66.67%)	0.591	0.442
	Female	4(22.22%)	7(33.33%)		
Age group	≤20	0(0%)	0(0%)	0.022	0.882
	21–64	9(50.00%)	10(47.62%)		
	≥65	9(50.00%)	11(52.38%)		
Specimen sources	Sputum	12(66.67%)	14(66.67%)		0.955
	Whole blood	1(5.56%)	2(9.52%)		
	Thoracic (abdominal) drainage fluid	1(5.56%)	2(9.52%)		
	Bronchial lavage fluid	1(5.56%)	0(0%)		
	Cerebrospinal fluid	1(5.56%)	0(0%)		
	Secreta	1(5.56%)	1(4.76%)		
	Urine	1(5.56%)	2(9.52%)		
No. of total	Total	18	21		

For Specimen sources, Fisher’s exact test was used due to 12 cells with expected counts <5, which provides exact *p*-values without relying on chi-square approximation (thus no chi-square statistic is reported).

### Phylogenetic analysis of *Acinetobacter baumannii* isolates

**Figure 1:**
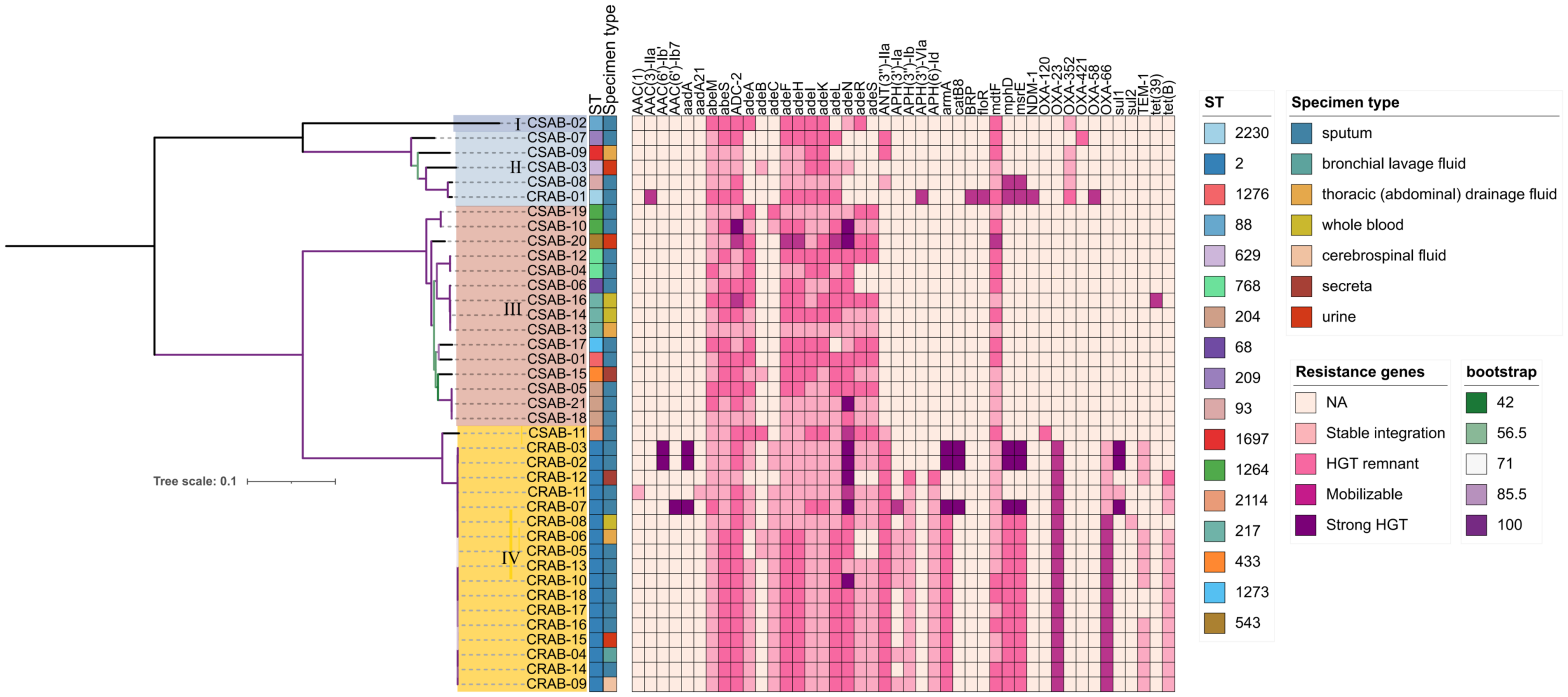
Phylogenetic analysis of *Acinetobacter baumannii* isolates. Antimicrobial microbial resistance (AMR) gene mobility status was inferred based on scaffold type (plasmid vs. chromosome) and presence of mobile genetic elements. Genes located on plasmids with Mobile Genetic Elements (MGEs) were classified as strong HGT evidence, those on plasmids without MGEs as mobilizable, those on chromosomes with MGEs as HGT remnants, and those on chromosomes without MGEs as stably integrated. Color gradient represents the predicted acquisition pathway: darker hues denote higher likelihood of HGT, whereas lighter tones indicate vertical inheritance or gene absence.

**Figure 2:**
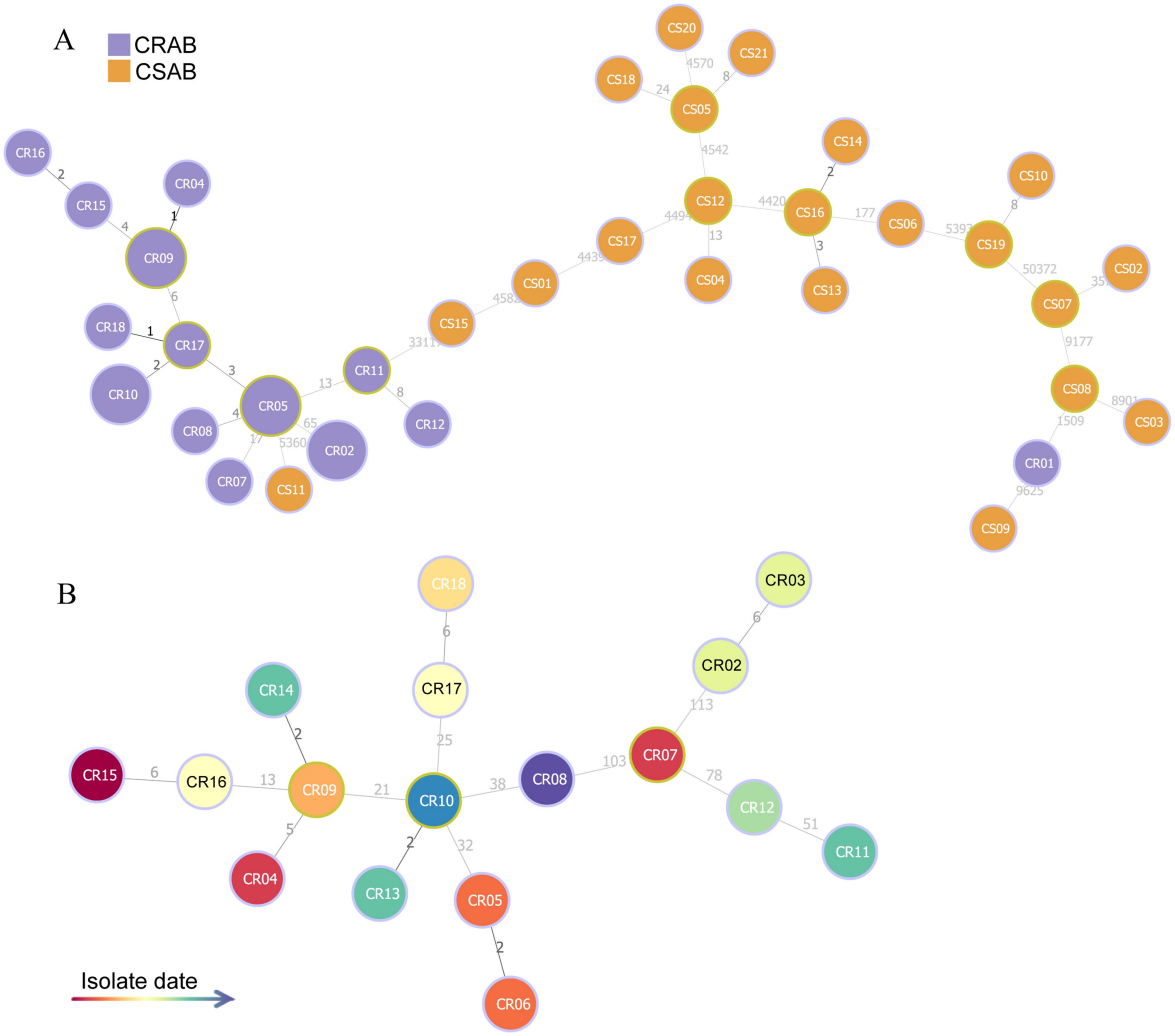
MST based on core genome SNP data of 39 *A. baumannii* isolates. **(A)** MST reconstruction comparing CR-AB (purple nodes) and CS-AB (orange nodes) isolates. **(B)** Temporal phylogeny of ST2 isolates, with node coloration indicating isolation dates (gradient scale: red = earliest to blue = most recent).

Using whole-genome sequencing data, we reconstructed the phylogeny of 39 clinical *A. baumannii* isolates to determine their evolutionary relationships (Figure 1). Phylogenetic analysis revealed that all isolates clustered into four distinct major clades (Clades I–IV). Notably, CR-AB strains were predominantly enriched in Clade IV (94.44%, 17/18). MST analysis revealed two primary, well-differentiated clusters corresponding to CR-AB and CS-AB isolates (Figure 2A). The CR-AB isolates formed a tightly interconnected cluster on the left network quadrant, demonstrating high clonal relatedness. Notably, isolate CS11 appeared as an outlier within the CR-AB cluster, potentially indicating either recent carbapenem resistance loss or representing an intermediate evolutionary state. Substantial genetic divergence between clusters was evidenced by 33,117 core genome SNPs separating representative strains CR11 and CS15, strongly supporting independent evolutionary trajectories for these phenotypic groups. According to the Pasteur scheme, the predominant clone was identified as ST2, accounting for 94.44% (17/18) of the CR-AB isolates. The MST analysis of ST2 isolates clearly delineates the temporal dynamics within this dominant clonal lineage (Figure 2B). Early isolates (denoted by red and orange nodes), including CR15, CR04 and CR07, were distributed at peripheral positions of the network and exhibited relatively large genetic distances (e.g., 103 SNPs between CR07 and CR08), indicating early diversification of this lineage. The central nodes, particularly CR09 and CR07, displayed intermediate colors (yellow to light green), corresponding to isolates collected during the middle phase of the outbreak. These central nodes also demonstrated high connectivity, suggesting their potential roles as transmission hubs or founding strains of secondary transmission chains.

### Virulence genes of *Acinetobacter baumannii*

**Figure 3:**
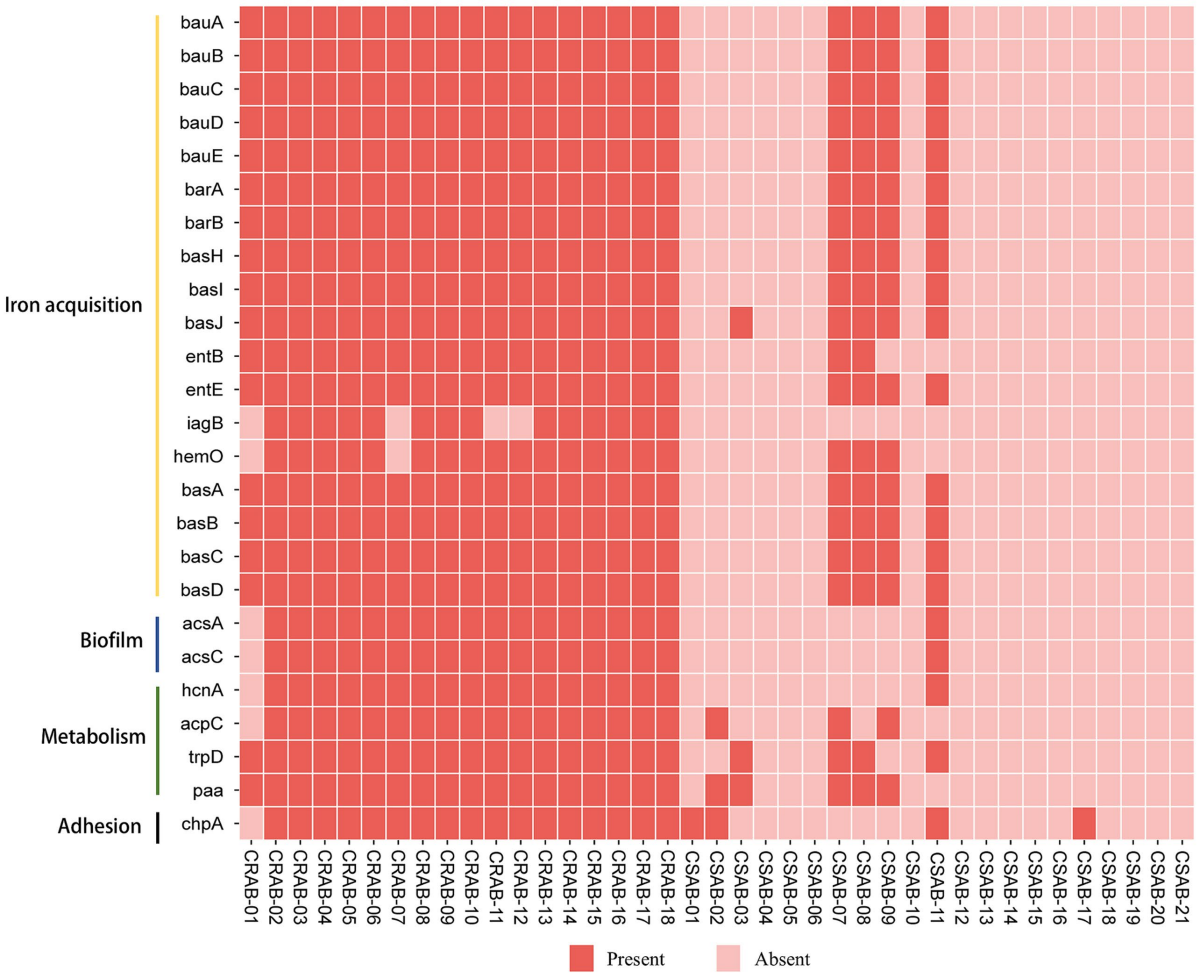
The distribution of virulence genes of *A. baumannii* in this study. The meaning of each column of colored squares is shown in the legend at the bottom.

**Figure 4:**
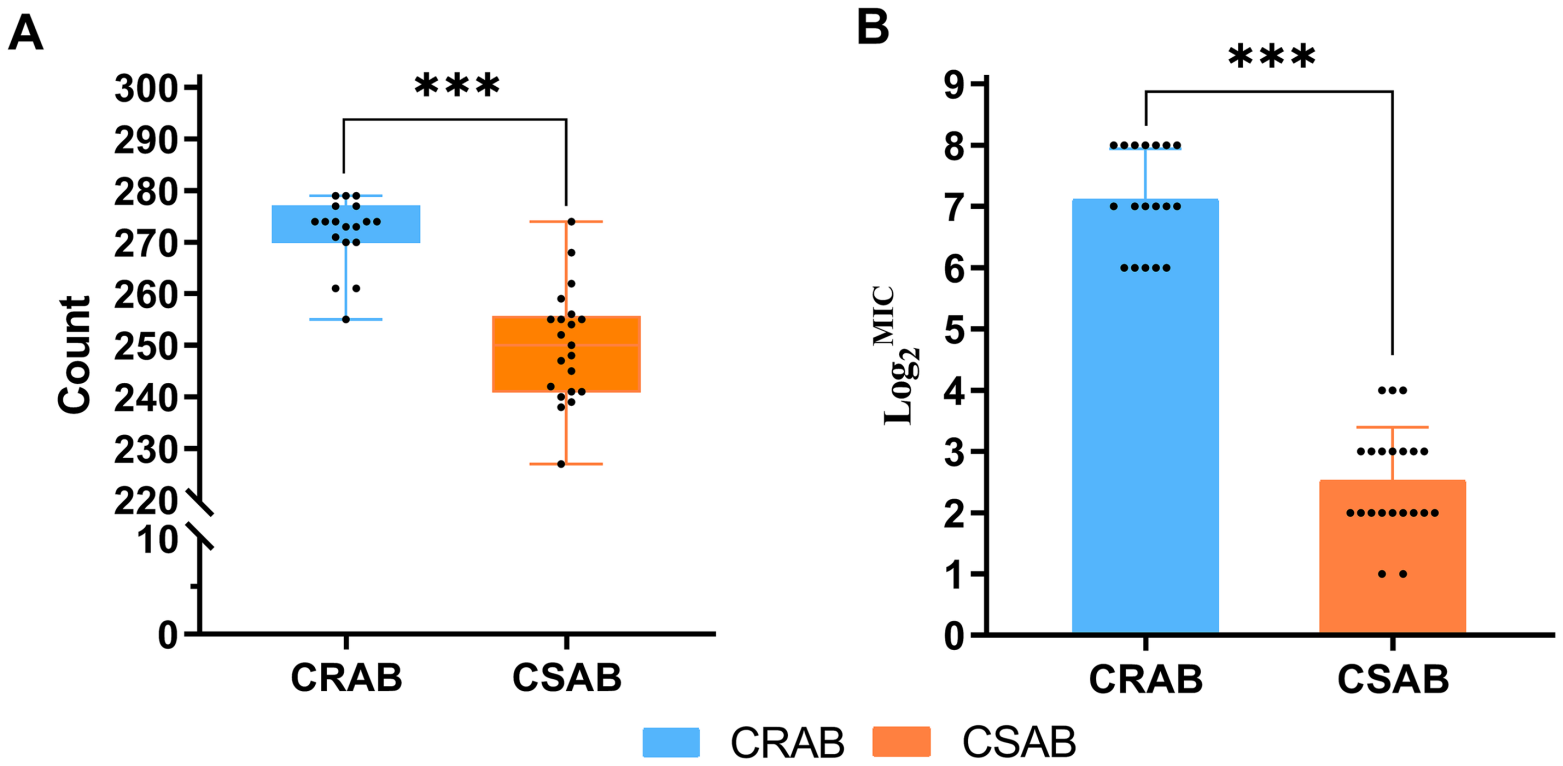
**(A)** Number of virulence genes carried by 39 strains of *A. baumannii* (CR-AB vs. CS-AB), Virulence gene count: CR-AB (272 ± 7) vs. CS-AB (250 ± 11). **(B)** MIC of Ceftazidime/Avibactam against 39 *A. baumannii* Isolates (CR-AB vs. CS-AB). *p < 0.05, **p < 0.01, ***p < 0.001.

The investigation of virulence genes revealed significant differences in the presence of virulence-associated genes among the 39 *A. baumannii* isolates (Figure 3). A comparative analysis between CR-AB and CS-AB isolates highlighted considerable disparities in the prevalence of key virulence genes across four distinct categories: iron acquisition systems (including *bauA, bauB, bauC, bauD, bauE, barA, barB, basH, basI, basJ, entB, entE, iagB, hemO, basA, basB, basC, basD*), biofilm formation (*acsA, ascC*), metabolic regulation (*hcnA, acpC, trpD, paa*), and adhesion-related genes (*chpA*), with statistically significant differences (*p* < 0.001) (Supplementary Table 2). Notably, CR-AB isolates exhibited a significantly higher prevalence of virulence genes within specific functional categories compared to CS-AB isolates. Moreover, CR-AB strains harbored a greater overall number of virulence genes (*p* < 0.001) (Figure 4A). This finding suggests that CR-AB strains may enhance their environmental adaptability and pathogenic potential through the acquisition of a broader spectrum of virulence factors. Specifically, the integrity and diversity of iron acquisition genes in CR-AB strains critically contribute to persistent host infection, enhancing pathogenicity and environmental survival.

### *Galleria mellonella* larva infection model experiment

**Figure 5:**
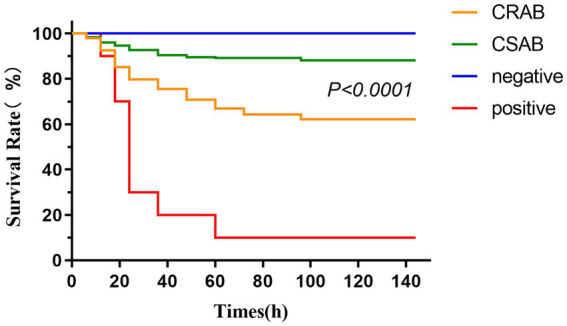
Survival curves of *Galleria mellonella* larvae in the infection model (CR-AB vs. CS-AB). Larval death was determined by turning black and not responding to mechanical stimuli.

We evaluated pathogenicity differences between CR-AB and CS-AB clinical isolates using the *G. mellonella* larva infection model (Figure 5). The experiment included three groups: (1) a negative control injected with saline, (2) a positive control receiving *A. baumannii* ATCC-AB5075, and (3) test groups infected with CR-AB or CS-AB isolates. The negative control maintained 100% larval survival, while the positive control showed only 10% survival. Notably, CR-AB infection resulted in significantly lower larval survival (60.56%) compared to CS-AB infection (87.62%) (*p* < 0.0001). These findings demonstrate that CR-AB isolates exhibit significantly greater pathogenicity than CS-AB isolates in this infection model.

### Antibiotic resistance genes of *Acinetobacter baumannii*

Our analysis of antibiotic resistance gene profiles revealed significant differences between CR-AB and CS-AB clinical isolates (Table 2 and Figure 1). CR-AB strains exhibited a highly conserved resistance gene profile, commonly carrying multiple resistance genes, including carbapenemase genes such as *bla*_OXA-23_ (94.44%, 17/18). Other resistance genes included β-lactam genes such as *bla*_OXA-66_ (94.44%, 17/18), and *bla*_TEM-1_ (66.67%, 12/18); aminoglycoside resistance genes *ANT(3″)-IIa* (94.44%, 17/18), *armA* (83.33%, 15/18), *APH(3″)-Ib* (83.33%, 15/18), *APH(6)-Id* (83.33%, 15/18), and *APH(3′)-Ia* (38.89%, 7/18); tetracycline resistance gene *tetB* (77.78%, 14/18); macrolide resistance genes *mphD* (88.89%, 16/18) and *msrE* (88.89%, 16/18); and efflux pump gene *adeC* (88.89%, 16/18), forming a typical pan-resistance gene profile. In contrast, CS-AB isolates only sporadically contained resistance genes from individual classes, with significantly lower carrying rates and gene diversity compared to CR-AB isolates.

**Table 2:** Resistance genes with significant differences between CR-AB and CS-AB.

Resistance Genes	CR-AB (*n* = 18)		CS-AB (*n* = 21)		*χ* ^2^	*P-*value
	Strains (n)	Percentage (%)	Strains (n)	Percentage (%)		
*bla*_OXA-23_	17	94.44	0	0.00	35.159	<0.001
*bla*_OXA-66_	17	94.44	0	0.00	35.159	<0.001
*bla*_TEM-1_	12	66.67	0	0.00	20.222	<0.001
*armA*	15	83.33	0	0.00	28.438	<0.001
*ANT(3″)-IIa*	17	94.44	4	19.05	22.170	<0.001
*APH(3″)-Ib*	15	83.33	0	0.00	28.438	<0.001
*APH(6)-Id*	15	83.33	0	0.00	28.438	<0.001
*APH(3′)-Ia*	7	38.89	0	0.00	–	0.002
*mphD*	16	88.89	1	4.76	27.897	<0.001
*msrE*	16	88.89	1	4.76	27.897	<0.001
*adeC*	16	88.89	2	9.52	24.565	<0.001
*tetB*	14	77.78	0	0.00	25.480	<0.001

For APH (3′)-Ia, Fisher’s exact test was used due to two cells with expected counts <5, which provides exact *p*-values without relying on chi-square approximation (thus no chi-square statistic is reported).

### Antimicrobial susceptibility of *Acinetobacter baumannii*

The results of the antimicrobial susceptibility tests revealed significant differences in the resistance profiles between CR-AB and CS-AB isolates (Table 3). CR-AB isolates demonstrated multidrug resistance, showing complete resistance to β-lactam antibiotics, including ceftazidime, cefepime, ceftriaxone, imipenem, and ampicillin/sulbactam, with a resistance rate of 100% (18/18). The resistance rates to aminoglycosides were as follows: tobramycin at 83.33% (15/18), gentamicin at 72.22% (13/18), and amikacin at 22.22% (4/18), with the relatively low resistance to amikacin indicating its potential as a limited option for empirical clinical treatment. The resistance rate to quinolones, specifically levofloxacin, was 88.89% (16/18), while the lowest resistance rate was observed for trimethoprim-sulfamethoxazole at 22.22% (4/18). In contrast, CS-AB isolates were fully susceptible to all the aforementioned antimicrobial agents, with a resistance rate of 0% (0/21).

**Table 3:** The resistance rates of CR-AB group and CS-AB group to clinical commonly used antibiotics.

Antibiotics	MIC Breakpoints (μg/mL)			CRAB (*n* = 18)		CSAB (*n* = 21)		*χ* ^2^	*P-*value
	*S* ^a^	*I* ^a^	*R* ^a^	*n* ^b^	%	*n* ^b^	%		
Ceftazidime	≤8	16	≥32	18	100.00	0	0.00	39.000	<0.001
Cefepime	≤8	16	≥32	18	100.00	0	0.00	39.000	<0.001
Ceftriaxone	≤8	16–32	≥64	18	100.00	0	0.00	39.000	<0.001
Imipenem	≤2	4	≥8	18	100.00	0	0.00	39.000	<0.001
Ampicillin/Sulbactam	≤8/4	16/8	≥32/16	18	100.00	0	0.00	39.000	<0.001
Amikacin	≤16	32	≥64	4	22.22	0	0.00	–	0.023
Gentamicin	≤4	8	≥16	13	72.22	0	0.00	22.750	<0.001
Tobramycin	≤4	8	≥16	15	83.33	0	0.00	28.438	<0.001
Sulfamethoxazole Trimethoprim	≤2/38	–	≥4/76	4	22.22	0	0.00	–	0.023
Levofloxacin	≤2	4	≥8	16	88.89	0	0.00	31.652	<0.001

^a^CLSI reference standard (CLSI, 2023); S, susceptible; I, intermediate susceptibility; R, resistant; ^b^*n*, Number of resistant strains. For Amikacin and Sulfamethoxazole-Trimethoprim, Fisher’s exact test was used due to two cells with expected counts <5, which provides exact *p*-values without relying on chi-square approximation (thus no chi-square statistic is reported).

**Figure 6:**
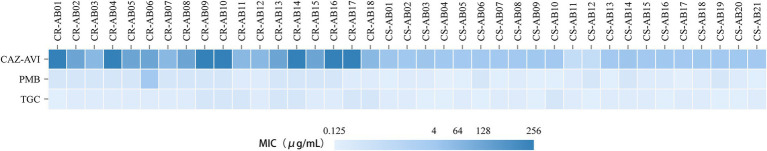
MIC distributions of polymyxin B, tigecycline, and ceftazidime/avibactam against the 39 *A. baumannii* isolates in this study.

The results of the antimicrobial susceptibility testing indicated that among the 39 clinical isolates of *A. baumannii*, the resistance rate to polymyxin B was 2.56% (1/39), all strains were sensitive to tigecycline (Table 4 and Figure 6). The MIC determination for polymyxin B revealed a range from 0.125 μg/mL to 4 μg/mL, with MIC50 and MIC90 values of 0.5 μg/mL and 1 μg/mL, respectively. For tigecycline, the MIC range was 0.25 μg/mL to 1 μg/mL, with MIC50 and MIC90 values also at 0.5 μg/mL and 1 μg/mL, respectively. The MIC distribution for ceftazidime/avibactam demonstrated considerable inter-group variability, with an overall MIC range spanning from 2/4 μg/mL to 256/4 μg/mL, and MIC50 and MIC90 values of 16/4 μg/mL and 256/4 μg/mL, respectively. Notably, the MIC range for CR-AB strains (64/4 μg/mL to 256/4 μg/mL) was significantly higher than that observed for CS-AB strains (2/4 μg/mL to 16/4 μg/mL) (*p* < 0.001) (Figure 4B). Comparative analysis of MIC50 and MIC90 values revealed that the MIC50 for CR-AB strains (128/4 μg/mL) was significantly elevated, being 32-fold higher than that for CS-AB strains (4/4 μg/mL), while the MIC90 for CR-AB strains (256/4 μg/mL) was 16 times greater than that for CS-AB strains (16/4 μg/mL).

**Table 4:** Antimicrobial susceptibility of *Acinetobacter baumannii.*

Antibiotics	MIC Breakpoints (μg/mL)			MIC range (μg/mL)	MIC50 (μg/mL)	MIC90 (μg/mL)	*n* (%)
	*S*	*I*	*R*				
Polymyxin B	–	≤2	≥4	0.125 ~ 4	0.5	1	1(2.44)
Tigecycline	≤2	4	≥8	0.25 ~ 1	0.5	1	0(0.00)
Ceftazidime/Avibactam		-		2/4 ~ 256/4	16/4	256/4	–

*n*, number of resistant strains; %, percentage of resistant strains.

### The effect of the efflux pump inhibitor PAβN on the MIC of ceftazidime/avibactam

**Figure 7:**
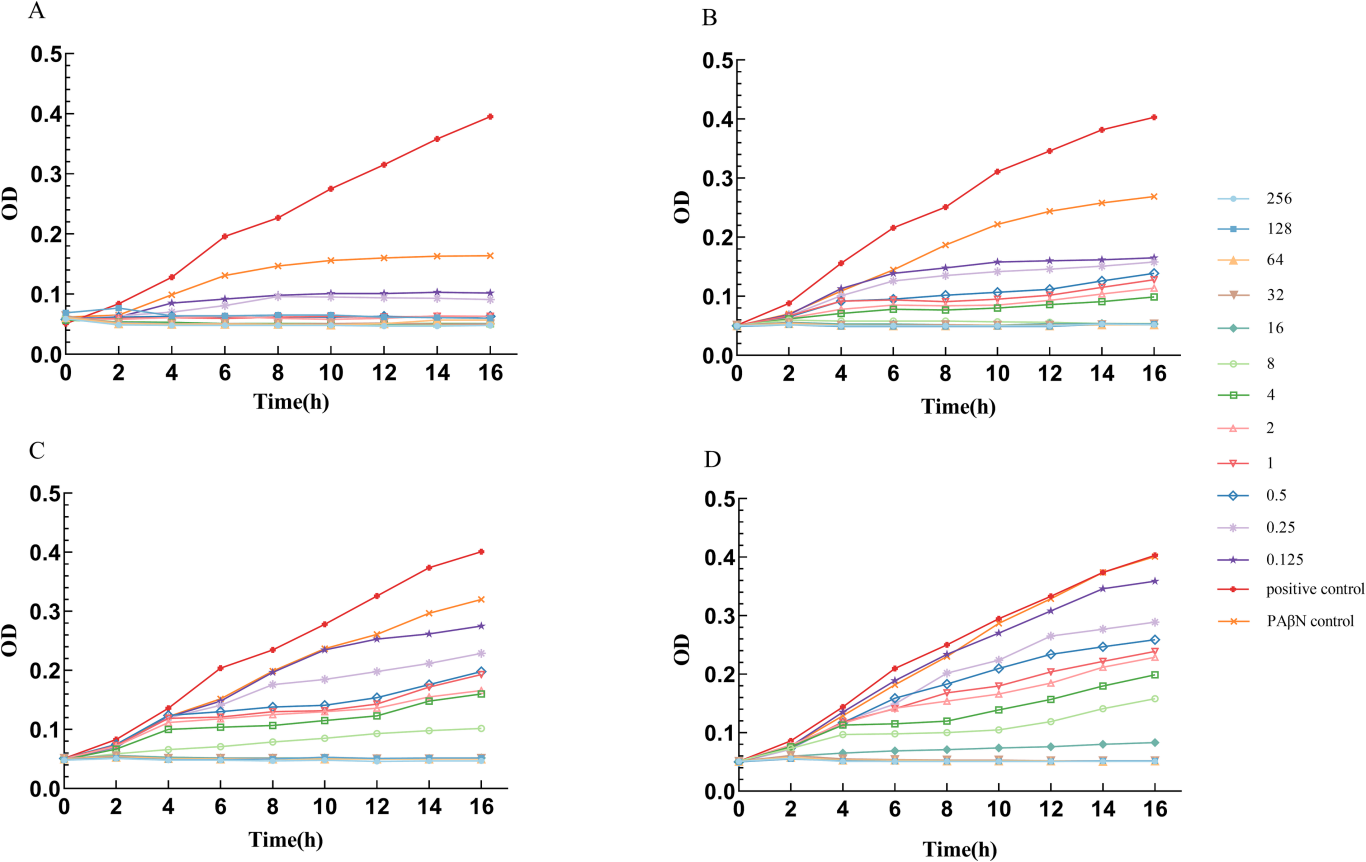
Growth curves of *A. baumannii* in different PAβN concentrations and different Ceftazidime/Avibactam concentrations. **(A)** PAβN 200 μg/mL, Ceftazidime in different concentrations (μg/ml). **(B)** PAβN 100 μg/mL, Ceftazidime in different concentrations (μg/ml). **(C)** PAβN 50 μg/mL, Ceftazidime in different concentrations (μg/ml). **(D)** PAβN 25 μg/mL, Ceftazidime in different concentrations (μg/ml). **(A–D)** The concentration of Avibactam was 4 μg/mL.

The efflux pump inhibition assay demonstrated a clear inverse correlation between PAβN concentration and ceftazidime/avibactam susceptibility (Figure 7). As the PAβN concentration decreased stepwise from 200 μg/mL to 25 μg/mL (200, 100, 50, and 25 μg/mL), we observed a corresponding dose-dependent increase in ceftazidime/avibactam MIC values (0.5, 8, 16, and 32 μg/mL, respectively). Notably, at a PAβN concentration of 50 μg/mL, the MIC of ceftazidime/avibactam was reduced by fourfold, from the original MIC of 64 μg/mL to 16 μg/mL. Additionally, PAβN exhibited a concentration-dependent inhibitory effect on bacterial growth, with its antimicrobial activity increasing in correlation with its concentration (Supplementary Table 3).

## Discussion

Research conducted by Huang et al. demonstrates that the detection rate of CR-AB in newly established hospital wards is generally low initially, but tends to increase over time as the patient population grows (Huang et al., 2013). Liu et al. (2022) documented a CR-AB detection rate of up to 77.00% in ICU environments, whereas the detection rate in our hospital’s ICU was 46.15%. This comparatively lower rate may be attributed to the fact that our ICU, inaugurated in 2016, is equipped with state-of-the-art medical facilities, implements stringent environmental disinfection protocols, and experiences relatively lower selective pressure from antimicrobial usage. Notably, the majority of CR-AB clinical isolates in this study were obtained from sputum samples (66.67%, 12/18), a distribution pattern that significantly diverges from the findings of Kahraman Kilbas (2023). This discrepancy may be related to the fact that all participants in this study were ICU patients, many of whom were subjected to mechanical ventilation with endotracheal intubation.

**Figure 8:**
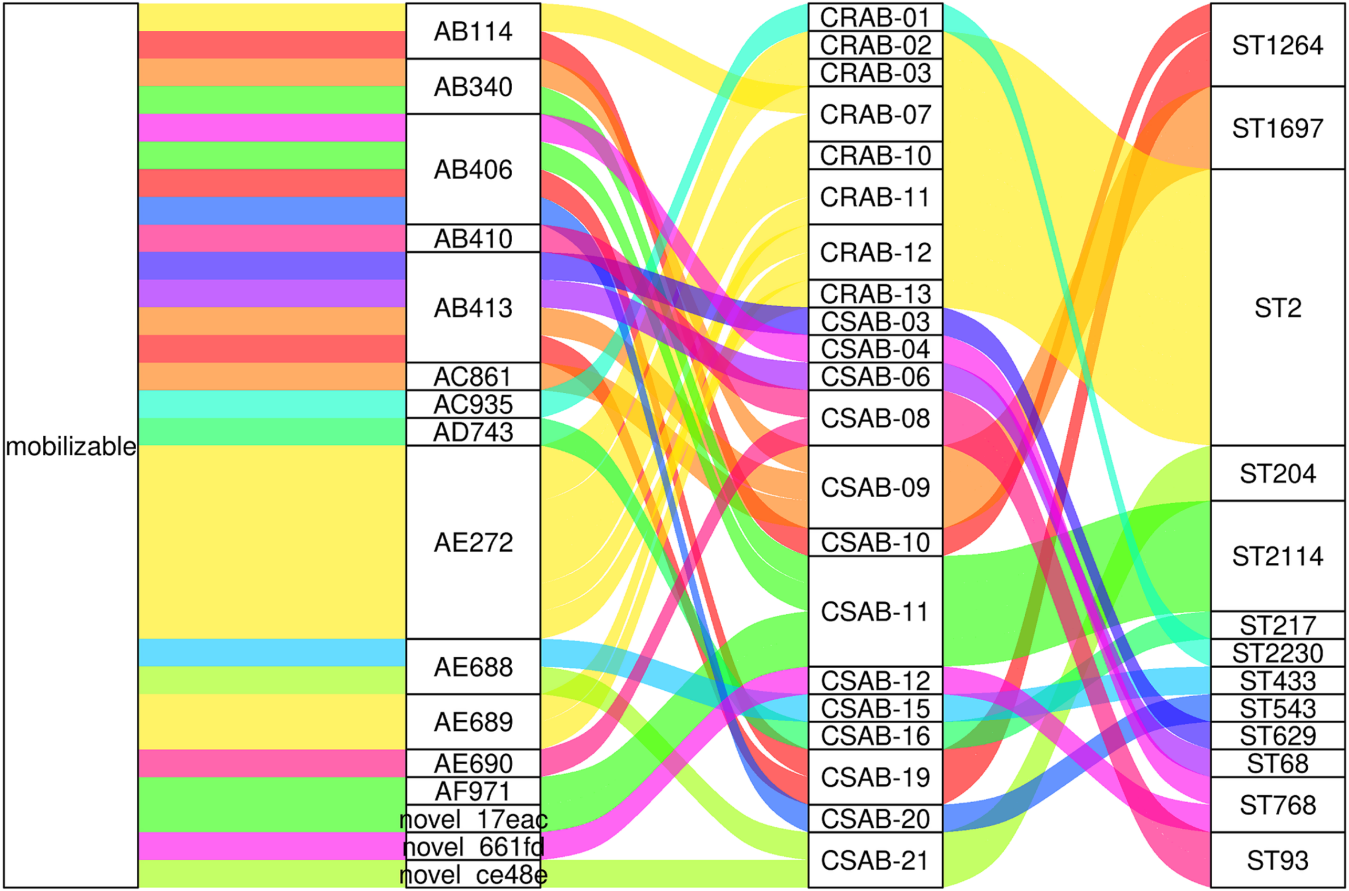
Mobilizable plasmids are widely shared across diverse ST types in *A. baumannii*. The diagram displays (from left to right): plasmid mobility status, plasmid cluster ID, isolate designation, and MLST sequence type.

Molecular typing utilizing the Pasteur scheme identified ST2 as the predominant type among CR-AB isolates, comprising 94.44% (17/18) of the samples. This sequence type is not only the most prevalent strain in China but also the most frequently reported in global surveillance efforts and various international databases (Hamidian and Nigro, 2019). ST2 is characterized by its robust biofilm formation capability, high serum resistance, and pathogenicity (Yu et al., 2021), alongside a higher prevalence of antibiotic resistance genes (Made Rai Dwitya et al., 2023). Surveillance data from Spain identified ST1 and ST2 as the predominant clonal phenotypes, representing the major multidrug-resistant (MDR) lineages (Cristina et al., 2024), ST2 and ST25 are widely disseminated in Thailand (Made Rai Dwitya et al., 2023). In contrast, South American studies revealed a distinct ST distribution pattern, where ST2 was notably absent, and ST1, ST79, and ST15 constituted a region-specific clonal complex (Barbara et al., 2022). Liu et al. (2024) demonstrated that ST164 represents an emerging high-risk lineage of global concern. Genome diversity analysis revealed the presence of 15 distinct ST types among the 21 CS-AB strains, whereas only 2 ST types were observed among the 18 CR-AB strains (Figure 1). These findings indicate that CS-AB strains exhibit greater genetic diversity (Chew et al., 2025), while CR-AB strains may be undergoing clonal expansion (Figure 8). Multiple plasmid clusters (e.g., AB114, AB340, and AB406) were identified in both CRAB and CSAB isolates, suggesting potential inter-lineage plasmid transfer. Notably, the plasmid cluster AE272 was the most widely distributed, being detected in multiple CRAB isolates. These isolates were predominantly associated with ST2, indicating that this plasmid cluster has spread extensively within the ST2 clonal background. Globally, the ST2 lineage—linked to carbapenem resistance—exhibited the highest diversity and the most abundant mobilizable plasmids, further supporting its role as both a reservoir and a vector for plasmid dissemination. In contrast, certain ST types (e.g., ST543, ST629) harbored only a limited number of unique plasmids, which may suggest lineage-specific plasmid restriction. Several plasmid clusters were shared across different ST types. For instance, plasmid AB413 was detected in isolates of ST629, ST68, and ST1697, implying recent horizontal transfer events. Similarly, plasmids AE688 and AE689 were identified in diverse CSAB isolates belonging to ST433 and ST204, further supporting the mobility of plasmids among genetically unrelated strains. Subsequent phylogenetic analysis categorized the CR-AB strains into two principal evolutionary clades: Clade-IV comprising 17 strains and Clade-II consisting of 1strains.

The findings of this study suggest that clinical isolates have developed virulence traits specifically targeting the host defense mechanisms (Chao et al., 2018; de Nies et al., 2021). Notably, there were significant differences (*p* < 0.001) between CR-AB and CS-AB isolates in the prevalence of virulence genes associated with iron acquisition systems, adhesion factors, biofilm formation, and metabolic pathways. This differential distribution of virulence genes may confer CR-AB strains with increased pathogenic potential, resulting in more severe clinical infections (Chao et al., 2018). This observation aligns with the markedly enhanced pathogenic phenotype of CR-AB observed in the *Galleria mellonella* larva infection model. Previous studies confirm virulence factor variation critically influences pathogenicity (Conde-Pérez et al., 2021). These findings enable targeted anti-virulence therapeutic development. Such strategies could serve as significant adjuncts to conventional antibiotic treatments (Trebosc et al., 2022).

The resistance genes present in CR-AB encompass those that confer resistance to carbapenems, β-lactams, aminoglycosides, macrolides, and tetracyclines. A strong correlation exists between the phenotypic resistance and genotypic profiles. In the current study, *bla*_OXA-23_ was identified in 94.44% (17/18) of CR-AB isolates, a prevalence rate consistent with that reported by Jiao et al. (2025). Previous surveillance data have indicated that *A. baumannii* remains highly susceptible to colistin (Li et al., 2019), a conclusion that matches the outcomes of this study. By analyzing the genomic localization of antibiotic resistance genes, we found that *bla*_OXA-23_ and *bla*_OXA-66_ in CRAB exhibit distinct hallmarks of horizontal gene transfer (HGT) (Figure 1 and Supplementary Table 4), suggesting that these genes may represent critical targets for transmission control interventions. The *tet (X)* gene, which elevates tigecycline MIC in *A. baumannii* (He et al., 2019), was absent in all isolates, explaining their tigecycline susceptibility. The sporadic resistance to these antibiotics in China likely arises from intrinsic mechanisms rather than plasmid-mediated spread. Key mechanisms include: (1) spontaneous mutations, (2) RND efflux pump overexpression, and (3) LOS modification gene inactivation, as opposed to *mcr-1*/*tet(X)*-carrying MDR plasmids. In this study, the resistance rate of *A. baumannii* to amikacin was determined to be 10.26% (4/39), which is significantly lower than the 40.90% reported by the CHINET 2023 national bacterial resistance surveillance network. This discrepancy may be attributed to reduced expression levels of resistance genes, such as those encoding aminoglycoside-modifying enzymes, among the isolates, as well as strain-specific variations in the distribution of capsular polysaccharide (CPS) serotypes (Chew et al., 2025).

The resistance mechanisms of *A. baumannii* to *β*-lactam antibiotics are intricate and multifaceted, primarily involving the production of β-lactamases and the overexpression of efflux pumps. In this study, we identified significant variations in the prevalence of resistance genes *bla*_TEM-1_, *bla*_OXA-66_, *bla*_OXA-23_, and *adeC* between CR-AB and CS-AB isolates. CR-AB strains demonstrated high levels of resistance to β-lactam antibiotics, including ceftazidime, ceftriaxone, and cefepime, whereas CS-AB strains exhibited high susceptibility, likely attributable to differences in the carriage rates of these resistance genes. Furthermore, we observed a statistically significant difference in the MIC of ceftazidime/avibactam between CR-AB and CS-AB isolates (*p* < 0.001) (Figure 4B). The *bla*_OXA-66_ variant is the most prevalent form of *bla*_OXA-51_ and is consistently identified in all ST2 isolates (Pasteur scheme) that also contain *bla*_OXA-23_ (Nurizati et al., 2024; Fernández-Cuenca et al., 2025). Avibactam is a novel non-β-lactam β-lactamase inhibitor. It shows potent activity against TEM-1 and OXA-51 enzymes (Ehmann et al., 2012; Juan Carlos et al., 2017; Compain et al., 2020), but has limited efficacy against some variants (Ling et al., 2024). Given that OXA-23 exhibits only weak hydrolytic activity against β-lactams, we suggest that the *adeC* gene could contribute to the resistance phenotype seen in these isolates. The *adeC* gene encodes a membrane channel component of the AdeABC we propose that the *adeC* gene may play a role in the resistance phenotype observed in these isolates efflux pump system, which is part of the RND family of efflux pumps (Neveen et al., 2024). The expression of AdeABC is regulated by the AdeRS two-component system (Stefanie et al., 2018; Yang et al., 2018). PAβN, a well-researched inhibitor of RND efflux pumps, is frequently used to investigate the function of efflux systems in antibiotic resistance (Fusté et al., 2013; Chalhoub et al., 2016; Shields et al., 2022). PAβN serves as a significant instrument in resistance reversal research; however, its clinical utility is constrained by pharmacokinetic limitations and potential cytotoxic effects. Our efflux pump inhibition assays largely validated the hypothesis: in the presence of 50 μg/mL PAβN, the MIC of ceftazidime/avibactam was reduced fourfold, from 64 μg/mL to 16 μg/mL. Further elevation of the inhibitor concentration to 100 μg/mL and 200 μg/mL led to eightfold (64 μg/mL to 8 μg/mL) and 128-fold (64 μg/mL to 0.5 μg/mL) reductions in MIC, respectively. Nonetheless, the exact molecular mechanism by which PAβN influences the AdeC protein remains elusive and necessitates further investigation. However, it is important to recognize that PAβN, while primarily characterized as an efflux pump inhibitor, may exert pleiotropic effects on bacterial cells. Beyond its canonical efflux inhibition activity, this compound could directly or indirectly modulate membrane permeability architecture (Al-Marzooq et al., 2023). This ancillary effect likely enhances intracellular antibiotic accumulation, thereby introducing a confounding variable in MIC interpretation where the relative contributions of efflux inhibition versus permeability enhancement become intrinsically coupled. Thus, the MIC modulations observed in our study probably represent an integrated response encompassing both efflux machinery blockade and membrane permeabilization. To dissect these interdependent mechanisms, future investigations could implement: (i) magnesium-supplemented media to maintain outer membrane stability (Ryan et al., 2013), or (ii) next-generation efflux inhibitors with improved target specificity.

## Conclusion

By integrating WGS with analyses of antimicrobial resistance and virulence phenotypes, this study offers the first comprehensive characterization of the molecular epidemiology of CR-AB and CS-AB isolates from ICU settings in the Ningbo region. The findings indicate that CR-AB isolates not only possess a broader antibiotic resistance profile but also exhibit significantly enhanced virulence in the *G. mellonella* infection model. This highlights the clinical significance of closely monitoring the transmission dynamics of such multidrug-resistant and highly virulent strains. Therefore, proactive observation and improved infection control practices are suggested to lessen the burden of hospital-acquired infections from these pathogens. To further validate our current findings, subsequent investigations will employ the following approaches: First, quantitative real-time PCR (qPCR) will be employed to precisely measure the expression levels of virulence-associated genes in CR-AB clinical isolates. Furthermore, we will establish a murine infection model to systematically evaluate CR-AB pathogenicity, with particular focus on monitoring the kinetic profiles of key proinflammatory cytokines (IL-6, TNF-*α*, and IL-1β), lactate, short-chain fatty acids (acetate, propionate, and butyrate) and LPS levels in serum following infection. Efflux pump inhibition assays demonstrated that PAβN markedly enhanced the susceptibility of CR-AB to ceftazidime/avibactam, underscoring the pivotal role of efflux mechanisms in resistance mediation. Phylogenetic analysis identified ST2 according to the Pasteur scheme as the predominant clonal lineage among local CR-AB isolates. This study provides critical scientific data for the prevention and management of hypervirulent CR-AB in ICU settings, through comprehensive investigations from both molecular epidemiological and drug resistance mechanism perspectives. The observed potentiating effects of efflux pump inhibitors offer a promising therapeutic target for optimizing treatment regimens against CR-AB infections, particularly in the context of combination therapy strategies. Future investigations should focus on elucidating the regulatory networks governing efflux pump expression and their potential co-evolutionary relationships with virulence factors, which would substantially advance our understanding of CR-AB pathogenesis. However, the pleiotropic effects of PAβN must be considered in data interpretation, as this compound may directly or indirectly modulate membrane permeability, thereby enhancing intracellular antibiotic accumulation. Consequently, the MIC alterations observed in this study likely represent combined effects of efflux inhibition and membrane permeabilization. To dissect these distinct mechanisms, subsequent studies could employ magnesium-supplemented media to maintain outer membrane stability or utilize next-generation efflux inhibitors with improved target specificity.

## Data Availability

The datasets presented in this study can be found in online repositories. The names of the repository/repositories and accession number(s) can be found at: https://www.ncbi.nlm.nih.gov/, BioProject accession number PRJNA1274604.
