# Supplementary material for: Molecular characterization of carbapenem-resistant and carbapenem-sensitive Acinetobacter baumannii isolates from an intensive care unit in Ningbo, China
Source: Front Microbiol. 2025 Sep 3;16:1646319. doi: 10.3389/fmicb.2025.1646319 (PMC12440978; doi:10.3389/fmicb.2025.1646319)
Supplement: Supplementary file 2 [file Table_2.docx]

| Virulence Genes | CR-AB (n=18) | | CS-AB(n=21) | | χ² | *P* value |
| --- | --- | --- | --- | --- | --- | --- |
|  | Strains(n) | Percentage (%) | Strains(n) | Percentage (%) |  |  |
| *bauA* | 18 | 100.00 | 4 | 19.05 | 25.831 | <0.001 |
| *bauB* | 18 | 100.00 | 4 | 19.05 | 25.831 | <0.001 |
| *bauC* | 18 | 100.00 | 4 | 19.05 | 25.831 | <0.001 |
| *bauD* | 18 | 100.00 | 4 | 19.05 | 25.831 | <0.001 |
| *bauE* | 18 | 100.00 | 4 | 19.05 | 25.831 | <0.001 |
| *barA* | 18 | 100.00 | 4 | 19.05 | 25.831 | <0.001 |
| *barB* | 18 | 100.00 | 4 | 19.05 | 25.831 | <0.001 |
| *basH* | 18 | 100.00 | 4 | 19.05 | 25.831 | <0.001 |
| *basI* | 18 | 100.00 | 4 | 19.05 | 25.831 | <0.001 |
| *basJ* | 18 | 100.00 | 5 | 23.81 | 23.255 | <0.001 |
| *entB* | 18 | 100.00 | 2 | 9.52 | 31.757 | <0.001 |
| *entE* | 18 | 100.00 | 4 | 19.05 | 25.831 | <0.001 |
| *iagB* | 14 | 77.78 | 0 | 0.00 | 25.480 | <0.001 |
| *hemO* | 16 | 88.89 | 3 | 14.29 | 21.592 | <0.001 |
| *basA* | 18 | 100.00 | 4 | 19.05 | 25.831 | <0.001 |
| *basB* | 18 | 100.00 | 4 | 19.05 | 25.831 | <0.001 |
| *basC* | 18 | 100.00 | 4 | 19.05 | 25.831 | <0.001 |
| *basD* | 18 | 100.00 | 4 | 19.05 | 25.831 | <0.001 |
| *acsA* | 17 | 94.44 | 1 | 4.76 | 31.368 | <0.001 |
| *ascC* | 17 | 94.44 | 1 | 4.76 | 31.368 | <0.001 |
| *hcnA* | 17 | 94.44 | 1 | 4.76 | 31.368 | <0.001 |
| *acpC* | 17 | 94.44 | 3 | 14.29 | 24.927 | <0.001 |
| *trpD* | 18 | 100.00 | 4 | 19.05 | 25.831 | <0.001 |
| *paa* | 18 | 100.00 | 5 | 23.81 | 23.255 | <0.001 |
| *chpA* | 17 | 94.44 | 4 | 19.05 | 22.170 | <0.001 |

Table S1 Virulence genes with significant differences between CR-AB and CS-AB
