# Supplementary figures and images for: Molecular characterization of carbapenem-resistant and carbapenem-sensitive Acinetobacter baumannii isolates from an intensive care unit in Ningbo, China
Source: Front Microbiol. 2025 Sep 3;16:1646319. doi: 10.3389/fmicb.2025.1646319 (PMC12440978; doi:10.3389/fmicb.2025.1646319)

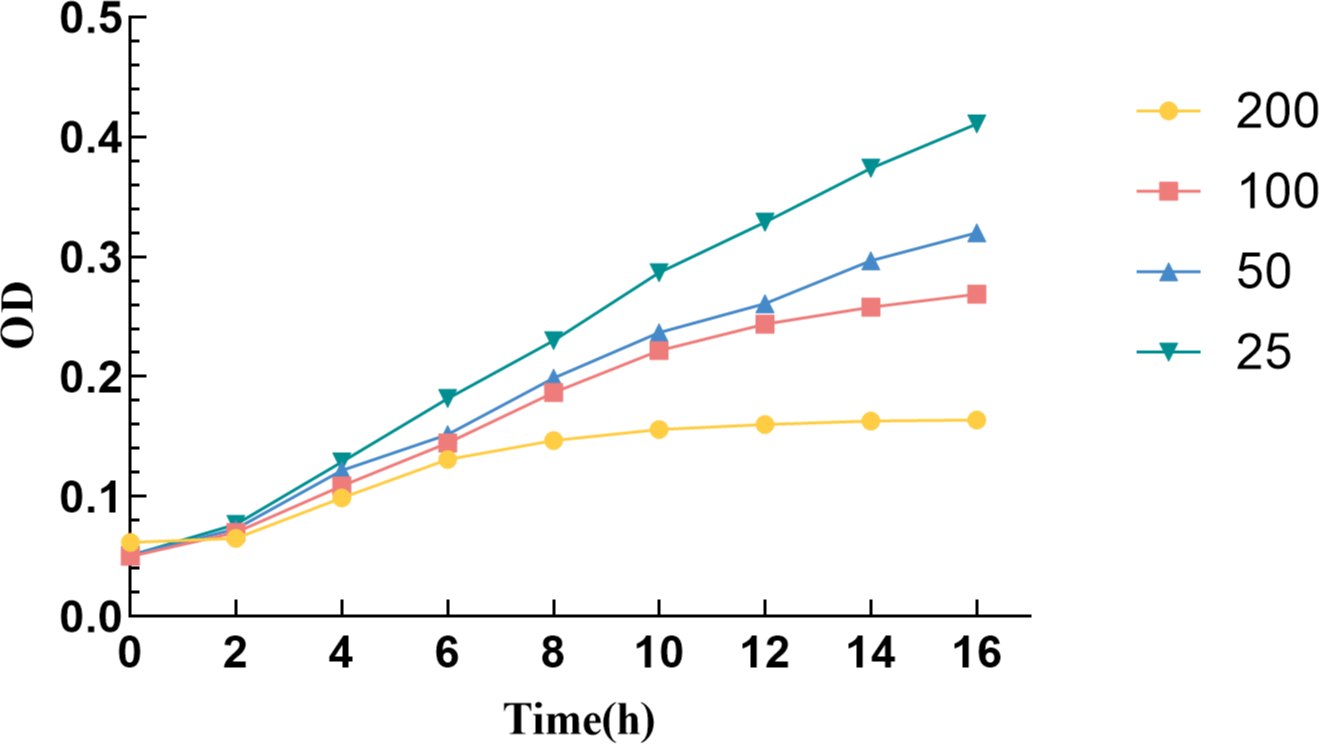


Figure S1. Growth curves of *Acinetobacter baumannii* in different PAβN concentrations (μg/ml).

Supplement: Supplementary file 3 [file Table_3.docx]
