# Supplementary material for: Molecular characterization of carbapenem-resistant and carbapenem-sensitive Acinetobacter baumannii isolates from an intensive care unit in Ningbo, China
Source: Front Microbiol. 2025 Sep 3;16:1646319. doi: 10.3389/fmicb.2025.1646319 (PMC12440978; doi:10.3389/fmicb.2025.1646319)
Supplement: Supplementary file 4 [file Table_4.docx]

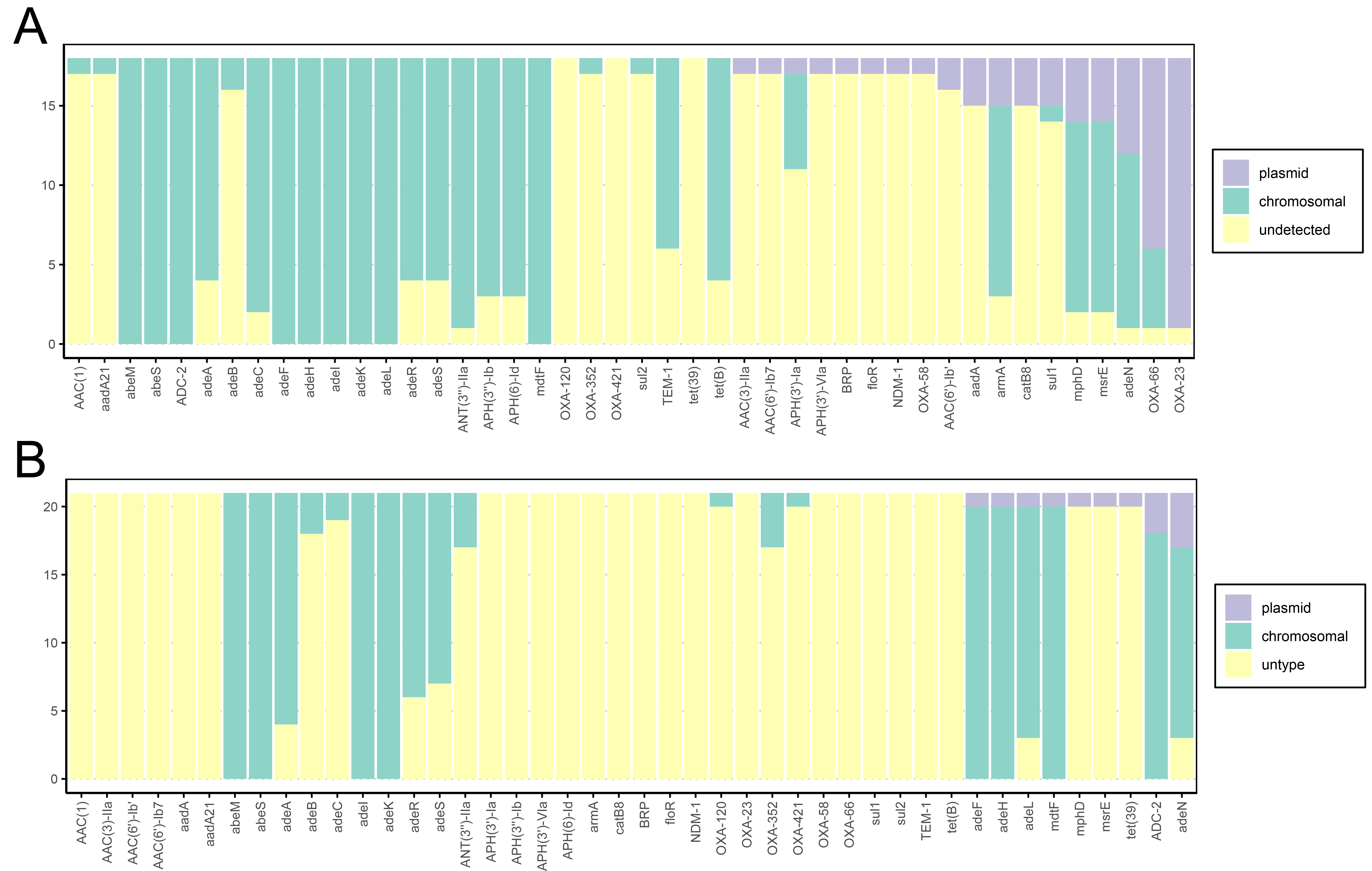


Figure S2. Genomic localization of key resistance genes in CR-AB and CS-AB isolates (plasmid-borne vs. chromosomal). (A) CR-AB. (B) CS-AB.
